# The uniqueness of morphological features of pure erythroid leukemia in myeloid neoplasm with erythroid predominance: A reassessment using criteria revised in the 2016 World Health Organization classification

**DOI:** 10.1371/journal.pone.0172029

**Published:** 2017-02-14

**Authors:** Po-Shen Ko, Yao-Chung Liu, Chiu-Mei Yeh, Jyh-Pyng Gau, Yuan-Bin Yu, Liang-Tsai Hsiao, Cheng-Hwai Tzeng, Po-Min Chen, Tzeon-Jye Chiou, Chia-Jen Liu, Jin-Hwang Liu

**Affiliations:** 1 Division of Hematology and Oncology, Department of Medicine, Taipei Veterans General Hospital, Taipei, Taiwan; 2 School of Medicine, National Yang-Ming University, Taipei, Taiwan; 3 Division of Transfusion Medicine, Department of Medicine, Taipei Veterans General Hospital, Taipei, Taiwan; 4 Institute of Public Health, National Yang-Ming University, Taipei, Taiwan; 5 Institute of Biopharmaceutical Sciences, National Yang-Ming University, Taipei, Taiwan; 6 Chong Hin Loon Memorial Cancer and Biotherapy Research Center, National Yang-Ming University, Taipei, Taiwan; European Institute of Oncology, ITALY

## Abstract

We reviewed 97 consecutive cases of myeloid neoplasm with erythroid predominance (MN-EP) between 2000 and 2015. Following 2016 WHO classification, MN-EP patients were classified into four groups. Eight pure erythroid leukemia (PEL) (including t-MN and AML-MRC morphologically fulfilled criteria for PEL) patients had dismal outcomes (median OS: 1 month) and showed more bone marrow fibrosis, worse performance status (PS) and higher serum lactate dehydrogenase (LDH) at diagnosis than the other groups. In the univariate analysis, risks of death in MN-EP patients included the morphologic features of PEL, very poor cytogenetic risk by IPSS-R, bone marrow fibrosis, leukocytosis, anemia, hypoalbuminemia, high LDH, and poor PS. In the multivariate analysis, independent predictors of death were morphologic features of PEL (adjusted hazards ratio [HR] 3.48, 95% confidence interval [CI] 1.24–9.74, *p* = 0.018), very poor cytogenetic risk by IPSS-R (adjusted HR 2.73, 95% CI 1.22–6.10, *p* = 0.015), hypoalbuminemia (< 3.7 g/dl) (adjusted HR 2.33, 95% CI 1.10–4.91, *p* = 0.026) and high serum LDH (≥ 250 U/L) (adjusted HR 2.36, 95% CI 1.28–4.36, *p* = 0.006). Poor or unfavorable risk in different cytogenetic risk systems independently predicted death and UKMRC-R was the best model.

## Introduction

Proliferation of erythroid precursors in bone marrow (BM) is not rare with myelodysplastic syndrome (MDS) or acute myeloid leukemia (AML). Among the myeloid neoplasms with variable proportion of erythroid precursors, erythroid predominance (MN-EP) (defined as erythroid precursors ≥ 50% of bone marrow nucleated cells) comprises up to 5% of AML and 15% of MDS cases [1–3]. The World Health Organization (WHO) 2008 classification of tumors of hematopoietic tissues classifies myeloid neoplasms with EP as five different entities: 1.) MDS, if BM myeloblasts are less than 20% of total nucleated cells and less than 20% of non-erythroid cells; 2.) therapy-related myeloid neoplasms (t-MN) if patients have undergone cytotoxic chemotherapy and/or radiotherapy; 3.) AML with myelodysplasia-related changes (AML-MRC) if meeting the criteria for AML-MRC; 4.) acute erythroid leukemia, if ≥ 20% BM myeloblasts of non-erythroid cells and not meeting the criteria of t-MN or AML-MRC [4].

In the WHO 2008 classification, acute erythroid leukemia, also called erythroleukemia [1] or DiGuglielmo’s syndrome [5, 6], is comprised of acute erythroleukemia, erythroid/myeloid type (AEL) and pure erythroid leukemia (PEL). It occurs more frequently in elderly patients and includes worse survival and a more frequent presence of poor risk cytogenetics than other AML subtypes [7]. Allogeneic hematopoietic stem cell transplantation (HSCT) has been reported to improve the dismal outcome of acute erythroid leukemia patients [8].

Acute erythroid leukemia definition has been debated regarding how to calculate BM myeloblasts and has been revised several times. Since the 2008 WHO classification, cases of morphologic PEL with selective proliferation of immature erythroblasts ≥ 80% of nucleated bone marrow cells fulfilled the criteria for t-MN or AML-MRC, were excluded from the diagnosis of PEL. However, the unique pathogenesis that leads to the selective erythroid proliferation and maturation arrest may infer the possible difference between morphologic PEL and t-MN or AML-MRC [9]. In the 2016 revision of the WHO classification, PEL is still viewed as a sole entity without any change in definition. AEL has been removed and is now recognized as AML, not otherwise specified (AML-NOS) if absolute myeloblasts ≥ 20% of all nucleated marrow cells or as MDS if absolute myeloblasts < 20% of all nucleated marrow cells but ≥ 20% of non-erythroid cells [10].

Such a change in the classification provides us the rationale to conduct this cohort study of patients with MN-EP. We report the impact on the survival outcome and the risk factors for mortality in these patients based on the 2016 WHO classification.

## Materials and methods

### Study population and data collection

The Institutional Review Board of Taipei Veterans General Hospital waived the need for written informed consent from the participants enrolled anonymously in the retrospective review of medical record and approved this study. This research conformed to the Helsinki Declaration and local legislation, and was approved by the Institutional Review Board of Taipei Veterans General Hospital. We consecutively enrolled patients newly diagnosed with MN-EP at Taipei Veterans General Hospital from January 1, 2000 to December 31, 2015. Cases that diagnosed as AML or MDS accompanied with erythroid predominance (defined as erythroid precursors ≥ 50% of bone marrow nucleated cells) were included. The patients demonstrated proliferation of BM erythroid precursor comprising ≥ 80% of nucleated bone marrow cells (including those fulfilled criteria for t-MN or AML-MRC) were classified into the group: PEL. Based on the 2016 WHO classification [10], other patients were further classified into three groups: 1.) AML-NOS, non-erythroid type (≥ 50% BM erythroid precursors, ≥ 20% myeloblasts of all cells in BM but not meeting t-MN or AML-MRC criteria); 2.) AML-MRC; 3.) MDS. We collected baseline general characteristics, clinicopathologic features and initial treatments by reviewing medical records. Follow-up information and survival data was continued until March 31, 2016. Reasons behind mortality were also reviewed for further analysis.

### Morphologic and pathologic evaluations

A peripheral blood smear, a bone marrow aspirate smear, and bone marrow biopsy at diagnosis were done for every patient in the cohort. Wright-Giemsa stains of bone marrow aspirate smears and hematoxylin-eosin–stained trephine specimens were reviewed by a hematopathologist and by at least one hematologist. If the final diagnosis was not consistent, another hematologist reviewed the marrow aspirate smears and biopsy specimen. By counting at least 500 nucleated cells of each bone marrow aspirate smear, percentages of bone marrow erythroid precursors of all nucleated cells, percentages of bone marrow myeloblasts of all nucleated cells, and percentages of bone marrow myeloblasts of non-erythroid cells were recorded. Percentages of bone marrow myeloblasts of all nucleated cells and of non-erythroid cells in bone marrow aspirate smears were also counted. Because the percentages of myeloblasts of PEL cases might be possibly overestimated based on the morphology of bone marrow aspirate smears, in which some immature erythroblasts might resemble myeloblasts of undifferentiated leukemia morphologically [11], the percentages of immunocytochemistry stained CD34 or MPO positive myeloblasts were required to be < 5% in PEL cases. Dysplastic percentages of precursors of myeloid, megakaryocytic, and erythroid precursors were also evaluated. By morphology, dysplasia recorded in Table 1 is defined as the presence of ≥ 10% dysplastic cells in the corresponding myeloid lineage. If morphologic dysplasia is used to define AML-MRC, the presence of ≥ 50% dysplastic cells in at least two cell lines is required. Gomori’s silver impregnation stain for reticulin was done to evaluate bone marrow fibrosis. The degree of bone marrow fibrosis was graded using a scale of 0–3 according to European bone marrow Fibrosis Network criteria. Patients whose grading belong to marrow fibrosis (MF)-1, 2, or 3 were considered as having bone marrow fibrosis [12].

**Table 1:** Baseline patient characteristics of myeloid neoplasm with erythroid predominance (MN-EP).

Characteristics	PEL *n* = 8	MDS *n* = 40	AML-MRC *n* = 30	AML-NOS (non- erythroid type) *n* = 19	Total *n = 97*	*P* value
	*n* (%)	*n* (%)	*n* (%)	*n* (%)	*n* (%)	
Median age, years (IQR)	68 (58–74)	68 (57–78)	75 (64–80)	72 (58–83)	72 (58–78)	0.299
≥ 60	6 (75.0)	28 (70.0)	24 (80.0)	14 (73.7)	72 (74.2)	0.825
< 60	2 (25.0)	12 (30.0)	6 (20.0)	5 (26.3)	25 (25.8)	
Sex						
Male	5 (62.5)	30 (75.0)	24 (80.0)	15 (79.0)	74 (76.3)	0.758
Female	3 (37.5)	10 (25.0)	6 (20.0)	4 (21.1)	23 (23.7)	
Cytogenetics, IPSS-R*						
Very good/good	2 (28.6)	13 (39.4)	8 (30.8)	15 (88.2)	38 (45.8)	0.005
Intermediate/poor	1 (14.3)	7 (21.2)	4 (15.4)	2 (11.8)	14 (16.9)	
Very poor	4 (57.1)	13 (39.4)	14 (53.9)	0 (0.0)	31 (37.4)	
Dyserythropoiesis^a^	3 (37.5)	31 (77.5)	24 (80.0)	6 (31.6)	64 (66.0)	< 0.001
Dysmegakaryopoiesis^a^	0 (0)	24 (60.0)	15 (50.0)	1 (5.3)	40 (41.2)	< 0.001
Bone marrow fibrosis^b^	3 (37.5)	6 (15.0)	1 (3.3)	(0.0)	10 (10.3)	0.011
HSCT	1 (12.5)	5 (12.5)	1 (3.3)	3 (15.8)	10 (10.3)	0.485
Laboratory data, median (IQR)						
WBC, 10^9^ /L	1.71 (1.25–5.08)	2.01 (1.46–3.18)	1.54 (1.13–2.71)	1.75 (1.29–2.51)	1.8 (1.32–2.83)	0.377
ANC, 10^9^/L	0.87 (0.54–2.52)	0.67 (0.36–1.13)	0.46(0.11–0.97)	0.50 (0.19–0.75)	0.56 (0.28–1.06)	0.237
Peripheral blasts, %	1.0 (0.0–6.75)	0.0 (0.0–5.8)	5.5 (2.0–12.0)	10.0 (6.0–19.0)	0 (0.0–6.0)	0.892
Hemoglobin, g/dl	7.52 (6.3–8.6)	7.5 (6.2–8.5)	7.7 (6.7–9.1)	8.0 (6.5–9.7)	7.6 (6.4–8.8)	0.874
Platelet, 10^9^/L	28.0 (10.3–78.0)	56.5 (29.5–98.0)	40.5 (30.0–63.0)	61.0 (34.0–87.0)	51.0 (29.0–88.0)	0.334
Bone marrow myeloblasts, %	0.0 (0.0–2.0)	10.0 (10.0–15.0)	20.0 (20.0–30.0)	20.0 (20.0–30.0)	15.0 (10.0–20.0)	< 0.001
Serum albumin, g/dl	3.2 (2.8–3.7)	3.6 (3.2–3.8)	3.5 (3.2–3.7)	3.8 (3.4–4.1)	3.6 (3.2–3.9)	0.145
Lactate dehydrogenase, U/L	578.0 (242.5.0–2597.5)	258.0 (204.0–386.0)	214.5 (169.0–424.0)	200.0 (166.0–248.0)	232.5 (172.0–413.0)	0.042
Ferritin, ng/mL	1,1102.5 (104.5–3,531.8)	537.0 (289.5–1,074.8)	582.3 (390.0–917.0)	487.0 (474.0–1,283.0)	573.7 (416.5–1,101.5)	0.329
ECOG performance status > 2	4 (50.0)	3 (7.5)	5 (16.7)	3 (15.8)	15 (15.5)	0.026
Treatment						
Curative chemotherapy	5 (62.5)	12 (30.0)	6 (20.0)	5 (26.3)	28 (28.9)	0.454
Non-curative therapy	1 (12.5)	9 (22.5)	7 (23.3)	4 (21.1)	21 (21.7)	
Supportive treatment	2 (25.0)	19 (47.5)	17 (56.7)	10 (52.6)	48 (49.5)	

MN-EP, myeloid neoplasm with erythroid predominance; PEL, pure erythroid leukemia; MDS, myelodysplastic syndrome; AML-MRC, acute myeloid leukemia, myelodysplasia-related changes; AML-NOS, acute myeloid leukemia, not otherwise specified; IQR, interquartile range; IPSS-R, Revised International Prognostic Scoring System; HSCT, Hematopoietic stem cell transplantation; WBC, white cell count; ANC, absolute neutrophil count; ECOG, the Eastern Cooperative Oncology Group performance score

* 14 cases had unavailable cytogenetic data

^a^ Dysplasia was defined as the presence of ≥10% dysplastic cells in the corresponding myeloid lineage.

^b^ MF-1, 2 or 3 based on European bone marrow Fibrosis Network criteria through Gomori’s silver impregnation stain for reticulin

### Cytogenetics

Conventional cytogenetic analysis was performed on G-banded metaphase cells prepared from bone marrow aspirate cultures using standard techniques during initial diagnosis. Twenty metaphase cells were analyzed and the results were reported using the International System for Human Cytogenetic Nomenclature. Because of coexistent features of MDS and AML in MN-EP patients, five different cytogenetic risk systems originally developed for AML or MDS were analyzed separately for the abilities of predicting overall survival. Based on the karyotype, patients were stratified to different cytogenetic risk groups according to United Kingdom Medical Research Council (UKMRC) criteria for AML [13], the revised United Medical Research Council (UKMRC-R) criteria for AML [14], the International Prognostic Scoring System (IPSS) for MDS, and the revised International Prognostic Scoring System (IPSS-R) [15,16]. In addition, the presence or absence of monosomal karyotype—which was defined as including at least two autosomal monosomies, or a single monosomy with additional structural abnormalities—was also used to stratified cases.

### Treatment

The initial diagnoses of cases enrolled were made before December 31, 2015 based on the 2008 WHO classification criteria, and most cases were initially diagnosed as acute erythroid leukemia or AML-MRC. Hence, curative chemotherapy included standard doses of induction chemotherapy, such as cytarabine with idarubicin or daunorubicin, or high-dose cytarabine with idarubicin or daunorubicin. Non-curative chemotherapy included low-dose cytarabine, or hypomethylating agents such as decitabine and 5-azacitidine. Allogeneic HSCT was arranged in refractory disease or in complete remission by clinicians’ judgment. Decisions about curative chemotherapy, non-curative chemotherapy or supportive care were made on an individual patient basis, at the discretion of the attending physician, and in agreement with the patient.

### Statistical analysis

Patients’ demographic and clinicopathological characteristics in the different groups are presented as the total number (*n*) and percentage (%). Cytogenetic risk groups were divided into tertiles according to cytogenetic risk classification, following IPSS-R criteria for MDS. We used chi-square tests or Fisher's exact tests to compare the categorical variables between groups. Data are presented as medians and interquartile ranges (IQR) for skewed data. The Kruskal-Wallis test was used for numerical comparison between more than two groups.

The primary endpoint was mortality. Survival probability was estimated using the Kaplan–Meier method; differences between groups were tested by log-rank test. The risk factors for mortality among these patients were calculated using a Cox proportional hazards model, and hazards ratios (HRs) and the 95% confidence intervals (CIs) were calculated. We used a multivariate Cox proportional hazards model to identify risk factors of mortality in patients with MN-EP while adjusting for possible independent confounding factors. All risk factors with a *p* < 0.1 in the univariate model were further entered into the multivariate analysis. Association of treatment with risk of mortality was assessed with Cox proportion hazards models, with treatment included as a time-dependent covariate.

Furthermore, we conducted a sensitivity analysis by using different definitions of cytogenetic risks according to IPSS, IPSS-R, UKMRC-R, UKMRC and monosomal karyotype. In the sensitivity analysis, we conducted the same statistical analysis on all study cohorts but used different definitions of cytogenetic risks to verify the relationship between cytogenetic risks and mortality. The corrected Akaike information criterion (AIC) and Bayesian information criterion (BIC) were used to determine which model was best supported by the data.

Data management and all statistical analysis were performed using SAS 9.3 software (SAS Institute Inc., Cary, NC) or STATA statistical software (version 12.1; StataCorp). All statistically significant levels were set at *p* < 0.05.

## Results

### Clinical characteristics of the study population

**Fig 1:**
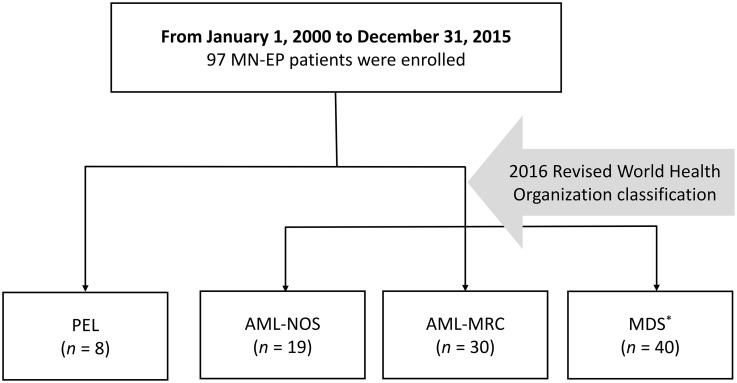
Patient selection. MN-EP, myeloid neoplasm with erythroid predominance; AML-NOS, acute myeloid leukemia, not otherwise specified; AML-MRC, acute myeloid leukemia, myelodysplasia-related changes; MDS, myelodysplastic syndrome. *Including refractory anemia with excess of blasts (RAEB)- 1, *n* = 2 and RAEB-2, *n* = 38.

**Fig 2:**
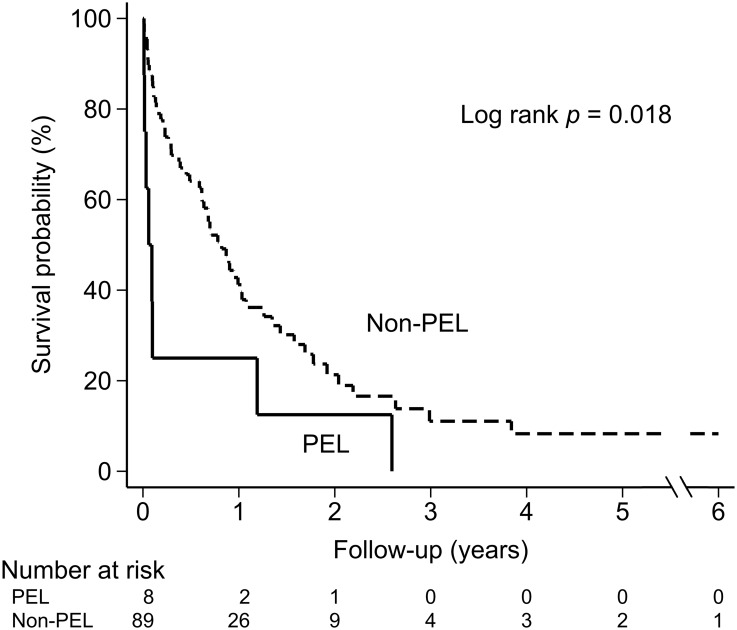
Survival curves of patients of myeloid neoplasm with erythroid predominance. PEL, Morphologic pure erythroid leukemia.

A total of 97 cases of MN-EP diagnosed consecutively from January 1, 2000 to December 31, 2015 were identified (S1 File). After reclassifying the cohort, there were eight cases of PEL, 19 cases of AML-NOS, 30 cases of AML-MRC, and 40 cases of MDS (Fig 1). In the PEL group, there was one case of a lung cancer patient receiving etoposide treatment one year before diagnosis of PEL, but no cases progressed from a prior MN. In the AML-MRC group, two cases of AML-MRC were based on preexisting MDS, 14 cases on cytogenetics, and 14 cases on morphologic dysplasia (≥ 50% dysplastic cells in at least two cell lines by BM morphology). In the patients with preexisting MDS, one received thalidomide, and one received close monitoring without exposure to hypomethylating agents which might increase erythroid cells in BM [17]. MN-EP affected older age groups, with a median age of 72 (range 23–94), and had a male predominance, with a ratio of 4:1. Cytopenia at diagnosis was common, and even pancytopenia occurred in 77 (79.4%) of the 97 patients. Only two patients presented with leukocytosis at diagnosis. The median overall survival (OS) of all patients was 8.7 months, with a median follow-up time of 4.9 months. The median OS of PEL patients was one month significantly lower than the 9.8 months seen with non-PEL patients (*p* = 0.018) (Fig 2).

The baseline clinical characteristics of the different four groups are shown in Table 1. PEL and AML-MRC were more associated with very poor cytogenetic risk, according to IPSS-R. There was less presence of dyserythropoiesis and dysmegakryopoiesis in the PEL and AML-NOS patients. A greater proportion of the PEL patients had bone marrow fibrosis. Although PEL patients had fewer bone marrow myeloblasts, they presented with higher serum lactate dehydrogenase (LDH) and poor Eastern Cooperative Oncology Group (ECOG) performance status.

### Risk factors of mortality

In the univariate analysis, risk of mortality in patients with MN-EP was significantly high in those diagnosed as PEL according to the 2016 WHO classification (HR 2.50, 95% CI 1.12–5.58, *p* = 0.019), cytogenetic risk of intermediate/poor risk (HR 2.12, 95% CI 0.99–4.54, *p* = 0.054) and very poor risk (HR 2.48, 95% CI 1.37–4.49, *p* = 0.025) based on IPSS-R, with bone marrow fibrosis (HR 2.44, 95% CI 1.15–5.17, *p* = 0.020), as well as those with initial white blood cell count (WBC) ≥ 1.1 × 10^9^ /L (HR 3.69, 95% CI 0.88–15.41, *p* = 0.074), hemoglobin < 8 g/dl (HR 1.83, 95% CI 1.09–3.08, *p* = 0.022), serum albumin < 3.7 g/dl (HR 2.75, 95% CI 1.62–4.67, *p* < 0.001), serum LDH ≥ 250 U/L (HR 2.39, 95% CI 1.46–3.92, *p* = 0.001), and ECOG > 2 (HR 2.36, 95% CI 1.18–4.75, *p* = 0.016). In the multivariate analysis, the independent significant predictors of mortality were PEL (adjusted HR 3.48, 95% CI 1.24–9.74, *p* = 0.018), very poor cytogenetic risk based on IPSS-R (adjusted HR 2.73, 95% CI 1.22–6.10, *p* = 0.015), serum albumin < 3.7 g/dl (adjusted HR 2.33, 95% CI 1.10–4.91, *p* = 0.026) and serum LDH ≥ 250 U/L (adjusted HR 2.36, 95% CI 1.28–4.36, *p* = 0.006). The analysis is detailed in Table 2.

**Table 2:** Analysis of risk factors for mortality in myeloid neoplasm with erythroid predominance.

Predictive variables	Univariate analysis		Multivariate analysis^c^	
	HR (95% CI)	*P* value	HR (95% CI)	*P* value
Group				
MDS	reference		reference	
PEL	2.50 (1.12–5.58)	0.019	3.48 (1.24–9.74)	0.018
AML-MRC	1.43 (0.80–2.55)	0.218	1.36 (0.67–2.73)	0.393
AML-NOS (non-erythroid type)	0.85 (0.42–1.72)	0.652	2.22 (0.90–5.50)	0.084
Age ≥ 60	1.10 (0.63–1.91)	0.745		
Sex (male)	0.89 (0.52–1.53)	0.664		
Cytogenetics, IPSS-R				
Very good/good	reference		reference	
Intermediate/poor	2.12 (0.99–4.54)	0.054	1.65 (0.70–3.87)	0.253
Very poor	2.48 (1.37–4.49)	0.003	2.73 (1.22–6.10)	0.015
Dyserythropoiesis^a^	1.49 (0.89–2.48)	0.130		
Dysmegakaryopoiesis^a^	1.14 (0.70–1.86)	0.611		
Bone marrow fibrosis^b^	2.44 (1.15–5.17)	0.020	1.60 (0.52–4.90)	0.409
Laboratory data				
WBC ≥ 1.1 × 10^9^ /L	3.69 (0.88–15.41)	0.074	3.36 (0.70–16.16)	0.131
ANC < 0.8 × 10^9^ /L	0.72 (0.44–1.18)	0.192		
Peripheral blasts ≥ 1%	1.33 (0.82–2.17)	0.254		
Hemoglobin < 8 g/dl	1.83 (1.09–3.08)	0.022	1.64 (0.90–3.01)	0.109
Platelet < 100,000 /μl	1.21 (0.66–2.23)	0.532		
Bone marrow blasts < 10%	1.70 (0.87–3.35)	0.122		
Serum albumin < 3.7 g/dl	2.75 (1.62–4.67)	< 0.001	2.33 (1.10–4.91)	0.026
Serum creatinine ≥ 2 mg/dl	0.99 (0.39–2.50)	0.985		
Lactate dehydrogenase ≥ 250 U/L	2.39 (1.46–3.92)	0.001	2.36 (1.28–4.36)	0.006
ECOG >2	2.36 (1.18–4.75)	0.016	1.61 (0.69–3.74)	0.270

HR, hazard ratio; CI, confidence interval; MN-EP, myeloid neoplasm with erythroid predominance; PEL, pure erythroid leukemia; MDS, myelodysplastic syndrome; AML-MRC, acute myeloid leukemia, myelodysplasia-related changes; AML-NOS, acute myeloid leukemia, not otherwise specified; ECOG, the Eastern Cooperative Oncology Group performance score

^a^ Dysplasia was defined as the presence of ≥10% dysplastic cells in the corresponding myeloid lineage.

^b^ MF-1, 2 or 3 based on European bone marrow Fibrosis Network criteria through Gomori’s silver impregnation stain for reticulin

^c^ All factors with *p* < 0.1 in the univariate analysis were included in the Cox multivariate analysis.

### Impact of different cytogenetic risk classification on mortality

**Fig 3:**
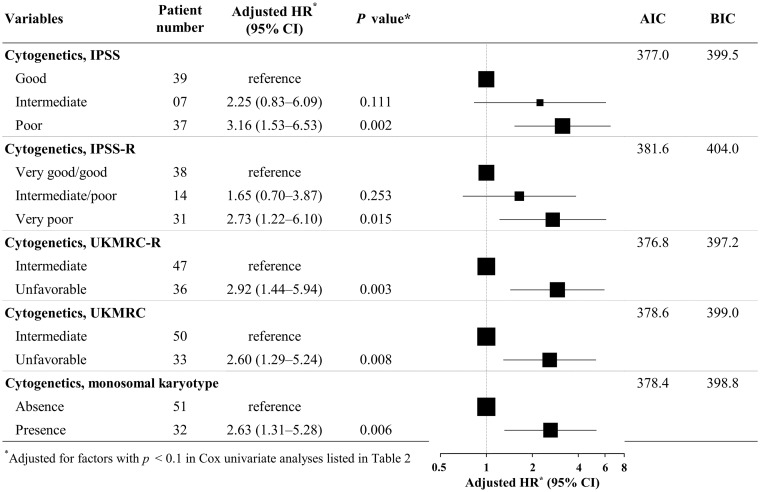
Impact of cytogenetic risk group on mortality in myeloid neoplasm with erythroid predominance. HR, hazards ratio; CI, confidence interval; AIC, Akaike information criterion; BIC, Bayesian information criterion. Two patients had very good cytogenetic results according to IPSS-R system.

There were 14 cases without available results of cytogenetic study. The majority of the patients had aberrant karyotypes (*n* = 49/83; 59.0%). To compare the predictive ability of different systems of cytogenetic risks, we applied five systems of cytogenetic risks to stratify the patients. After adjusting for factors with a *p* < 0.1 in the Cox univariate analysis in Table 2, the impacts of different cytogenetic risk classifications on mortality are illustrated in Fig 3. All cytogenetic risk classifications independently predicted mortality. UKMRC-R was the best model for predicting mortality, with the lowest AIC and BIC.

### Treatment and mortality

Allogeneic HSCT was administered to 10 patients (one PEL, one AML-MRC, three AML-NOS and five MDS cases), whose disease statuses at transplantation were refractory disease for one and complete remission for nine. As compared to supportive care, the mortality among MN-EP patients did not differ from that of the patients receiving HSCT, curative chemotherapy or non-curative chemotherapy. The analysis is shown in Table 3.

**Table 3:** Analyses of treatment for mortality in myeloid neoplasm with erythroid predominance.

Predictive variables	Univariate analysis		Multivariate analysis^b^	
	HR (95% CI)	*P* value	HR (95% CI)	*P* value
HSCT ^a^	0.47 (0.19–1.17)	0.103	0.60 (0.22–1.90)	0.303
Curative chemotherapy ^a^^,^ ^c^	0.90 (0.53–1.54)	0.710	1.18 (0.480.52–2.64)	0.665
Non-curative therapy ^a^	1.45 (0.81–2.60)	0.205	1.601.52 (0.69–3.80)	0.342

HR, hazard ratio; CI, confidence interval; HSCT, hematopoietic stem cell transplantation

^a^ Treatment was analyzed as a time-dependent covariate in the Cox regression model

^b^ Adjusted for factors with *p* < 0.1 in Cox univariate analyses listed in Table 2.

^c^ Curative chemotherapy included standard doses of induction chemotherapy, such as cytarabine with idarubicin or daunorubicin, or high-dose cytarabine with idarubicin or daunorubicin.

### Reasons for mortality

In this study, mortality occurred in 67 (69.1%) of the MN-EP patients, with a median survival of 5.5 months (range 0.1–46.8). Causes of mortality were infection in 59 (88.1%) of the deaths (including pneumonia in 24, bacteremia in 13, septic shock in 16, fungemia in three, and liver abscess in two cases), followed by three cases of sudden death, three cases of intracranial hemorrhage, one case of hemophagocytosis, and one case of liver failure. Among the different treatment groups, 21 of 28 patients (75.0%) receiving standard curative chemotherapy, 16 of 21 patients (76.2%) receiving non-curative chemotherapy, and 30 of 48 (62.5%) patients receiving supportive care died.

## Discussion

How to count percentages of BM myeloblasts (of total nucleated cells or non-erythroid cells) is no longer an issue after the revised 2016 WHO classification [10]. The present cohort study of MN-EP patients is the first to report clinicopathological characteristics, survival outcomes and risk factors of mortality for new entities originating from MN-EP after the revision of WHO classification. The patients enrolled into analysis were all from Taipei Veterans General Hospital, a tertiary medical center in northern Taiwan. The national center performed the first case of allogeneic bone marrow transplantation and the first case of allogeneic peripheral blood HSCT in Taiwan [18]. All diagnoses were made under stringent standards and patients were managed by hematological specialists based on optimal strategies, with care provided by experienced nurses. Herein, we demonstrate the uniqueness of PEL after affirming it as an independent risk for death in MN-EP. Additionally, the five different classifications of cytogenetic risk groups all show effectiveness independently in predicting survivals, of which UKMRC-R is the best system. Other significant risk factors for MN-EP are low serum albumin and high serum LDH.

PEL and acute erythroleukemia (erythroid/myeloid type) comprise acute erythroid leukemia in the 2008 WHO classification [4]. Despite many efforts to dissect MN-EP following several revised definitions of AEL, there was still much debate on how to classify these patients under the 2008 WHO classification [4]. In respect of erythroleukemia (erythroid/myeloid type), MN-EP not meeting criteria for t-MN or AML-MRC was arbitrarily considered as erythroleukemia (erythroid/myeloid type) if BM myeloblasts ≥ 20% of non-erythroid cells or MDS if BM myeloblasts < 20% of non-erythroid cells under the 2008 WHO classification. Therefore, the same MN-EP patient whose BM myeloblasts were around 20% of the non-erythroid cells may have had a different diagnosis resulting from a counting error. In addition, counting percentage of myeloblasts of non-erythroid cells in MDS and AEL cases was also reported to be of no prognostic significance [1, 19]. Moreover, erythroleukemia (erythroid/myeloid type) was similar with AML-MRC in aspects of cytogenetic changes, molecular findings and OS [20–23]. After FPS Santo *et al*. and RB Walter *et al*. both reported that AEL provided no prognostic information for cases of AML-NOS [3, 24], erythroleukemia (erythroid/myeloid type) was waived in the updated 2016 WHO classification and PEL remained as a sole specific entity with the same definition. PEL is well known for its very poor prognosis, and results from the proliferation of immature neoplastic erythroblasts rather than reactive left-shifted erythroblasts. It is also regarded as a different disease in clinical presentations, with different biological features and survival outcomes from the other AML types, which result from the proliferation of malignant myeloblasts. The erythroblasts in PEL are distributed in diffuse sheets rather than small clusters intermingled with other BM elements and show features, including smaller sizes, more dispersed chromatins, fewer but more prominent nucleoli, and frequently vacuolated cytoplasm than normal erythroblasts [25]. PEL could be de novo or from MDS but most bear complex karyotypes, and frequently chromosome 5 and/or chromosome abnormalities [26], but the latter are reclassified AML-MRC after the 2008 classification. However, the molecular characteristics of PEL are still needed to be studied in depth to determine a distinct, or shared, pathogenesis to AML-MRC or t-MN. Wong *et al*. [27] recommended that the distinct morphology of PEL should be recognized prudently for those of t-MN or AML-MRC with a note in parentheses, for example, AML-MRC (with PEL features). We demonstrate the uniqueness of PEL in the present study in regard to its’ correlation with survivals after being adjusted to take into consideration other significant risk factors in the univariate analysis.

After introduction of the 2016 WHO classification, Calvo *et al*., Wang *et al*., and Bennett *et al*. found that MDS patients previously diagnosed as AEL had similarly poor survival outcomes with those of high-risk MDS (i.e., MDS-RAEB in their study) [28–30]. In the present study, those patients with myeloblasts ≥ 20% of all nucleated cells previously diagnosed as AEL are now considered AML-NOS according to the 2016 WHO classification. There was no difference in survival outcome after adjustment for other risk factors for death in comparison with MDS. Most patients re-classified as MDS (*n* = 40) in the cohort belonged to poor IPSS-R risk groups (32 cases with available cytogenetic data, 23 in the very high-risk group, eight in the high-risk group, and one in the intermediate group; eight cases without available cytogenetic results, five in high-risk, and three in intermediate risk) might lead to the result. Although AML-MRC has been reported to include a worse outcome by comparison to AML-NOS [5], especially in patients carrying a mutated *ASXL1* gene [31], Devillier *et al*. pointed out the heterogeneity of AML-MRC, in which those with intermediate cytogenetic risk had non-inferior survival in patients with AML-NOS [32]. In the presence of EP, the prognoses of AML-MRC and AML-NOS were not different in our cohort.

According to our previous study, acute erythroid leukemia shares some features with MDS [33]. In the present report, we initially used IPSS-R as a variable in the multivariate analysis. Despite showing the effectiveness of IPSS-R, however, further analysis using AIC and BIC shows that UKMRC-R saw the best goodness of fit for prediction of overall survival. The original study group of UKMRC-R included de novo AML and secondary AML with myelodysplasia-related changes, which is similar to the present study [14]. Furthermore, Hasserjian’s study supported UKMRC-R as being the best model for cytogenetic classification in patients with acute erythroid leukemia [23]. Additionally, we show that monosomal karyotypes are prognostic in MN-EP, which has not been mentioned before. Our results need further verification.

One cytogenetic result of the eight PEL cases was lacking, and one showed normal karyotype while the other seven cases had complex karyotype, including five with chromosome 5 and/or 7 involved. In the past, PEL was rarely discussed in comparison with AEL, possibly due to its rarity. The largest cohort before ours included seven cases [21, 27, 34]. In the latest report comparing AML with EP by Wong *et al*., the median survival of 2.9 months in PEL was dismal, and a high incidence of adverse-risk cytogenetics was shown in PEL patients [27]. We tried to figure out the risk factors of death in PEL patients but no significant result surfaced due to a relatively small case number.

Serum LDH and albumin levels were reported as independent risk factors for mortality in AEL [24, 33]. Serum LDH is a well-established surrogate for tumor burden and has been shown prognostic value in AML and MDS [35, 36]. Hypoalbuminemia is an indicator of poor nutrition status and an adverse prognostic factor in multiple neoplastic diseases including AML [37, 38]. This study also shows the negative impacts of high LDH levels and hypoalbuminemia in the outcome.

The best strategy for treating acute erythroid leukemia in the past was to administer intensive induction chemotherapy, followed by allogeneic HSCT if complete remission was achieved [19, 33]. Despite recent reclassification shifting 40 MN-EP patients to MDS, most of them (*n* = 36, 90.0%) were IPSS-R high-risk and very high-risk. Allogeneic HSCT was assumed to be helpful in improving the outcome. However, most patients were not suitable for allogeneic HSCT due to old age. Three survivors after allogeneic HSCT were cases of MDS, all the other seven patients died of infection. Due to small numbers of HSCT and the fragility of the cohort, the benefits of allogeneic HSCT in AML-EP might be obscured.

Our study has some limitations. First, as a national medical center, this study might include patients with worse conditions at their time of visit, thereby negatively influencing the outcomes. Second, the proportion of de novo PEL patients seemed relative increased, which might be due to ethnic difference or coincidence. Third, mutation studies, including NPM1, CEBPA and TP53, at diagnosis of AML are not covered by National Health Insurance. The retrospective data about these important prognostic factors for further analysis was scarce. Finally, an unpredictable bias might result from different treatment strategies adopted by different attending physicians. The retrospective nature of the study also limits us to drawing firm conclusions, making totally exclusion of residual confounding factors in our study setting difficult.

## Conclusion

After the 2016 WHO classification was published, only PEL were kept as belonging to neoplasms originated from neoplastic erythroid lineage. This retrospective cohort study reports the clinical features, biological data and the survival outcome of patients with MN-EP and identified PEL group, very poor cytogenetic risk group of IPSS-R, high serum LDH and low serum albumin as independent predictors for death. Cytogenetic risks groups determined by UKMRC-R showed the best ability to predict the survival outcome of MN-EP patients. The outcome of PEL was independently much worse.

## References

[1] Wang SA, Tang G, Fadare O, Hao S, Raza A, Woda B, et al Erythroid-predominant myelodysplastic syndromes: enumeration of blasts from nonerythroid rather than total marrow cells provides superior risk stratification. Mod Pathol 2008;21:1394–1402. 10.1038/modpathol.2008.142 18839018

[2] Bennett JM, Begg CB. Eastern Cooperative Oncology Group study of the cytochemistry of adult acute myeloid leukemia by correlation of subtypes with response and survival. Cancer Res 1981;41:4833–4837. 6945906

[3] Walter RB, Othus M, Burnett AK, Löwenberg B, Kantarjian HM, Ossenkoppele GJ, et al Significance of FAB subclassification of "acute myeloid leukemia, NOS" in the 2008 WHO classification: analysis of 5848 newly diagnosed patients. Blood 2013;121:2424–2431. 10.1182/blood-2012-10-462440 23325837 PMC3612855

[4] Swerdlow SH, Campo E, Harris NL. WHO Classification of Tumours of Haematopoietic and Lymphoid Tissues. Lyon, France: IARC Press 2008.

[5] Di Guglielmo G. Ricerche di ematologia. Folia Med (Plovdiv) 1917;3:386–396.

[6] Dameshek W, Baldini M. The Di Guglielmo syndrome. Blood 1958;13:192–194. 13510296

[7] Olopade OI, Thangavelu M, Larson RA, Mick R, Kowal-Vern A, Schumacher HR, et al Clinical, morphologic, and cytogenetic characteristics of 26 patients with acute erythroblastic leukemia. Blood 1992;80:2873–2882. 1450412

[8] Fouillard L, Labopin M, Gorin NC, Polge E, Prentice HG, Meloni G, et al Hematopoietic stem cell transplantation for de novo erythroleukemia: a study of the European Group for Blood and Marrow Transplantation (EBMT). Blood 2002;100:3135–3140. 10.1182/blood.V100.9.3135 12384410

[9] Wong E, Juneja S. Acute myeloid leukaemia and myelodysplastic syndromes with 50% or greater erythroblasts: a diagnostic conundrum. Pathology. 2015;47(4):289–93. 10.1097/PAT.0000000000000244 25938365

[10] Arber DA, Orazi A, Hasserjian R, Thiele J, Borowitz MJ, Le Beau MM, et al The 2016 revision to the World Health Organization classification of myeloid neoplasms and acute leukemia. Blood 2016;127:2391–2405. 10.1182/blood-2016-03-643544 27069254

[11] Mihova D, Zhang L. Acute erythroid leukemia: a review. N Am J Med Sci, 2012;5:110–8.

[12] Thiele J, Kvasnicka HM, Facchetti F, Franco V, van der Walt J, Orazi A. European Consensus On Grading Bone Marrow Fibrosis And Assessment Of Cellularity. Haematologica 2005;90:1128–1132 16079113

[13] Grimwade D, Walker H, Oliver F, Wheatley K, Harrison C, Harrison G, et al The importance of diagnostic cytogenetics on outcome in AML: analysis of 1,612 patients entered into the MRC AML 10 trial. The Medical Research Council Adult and Children's Leukaemia Working Parties. Blood 1998;92:2322–2333. 9746770

[14] Grimwade D, Hills RK, Moorman AV, Walker H, Chatters S, Goldstone AH, et al Refinement of cytogenetic classification in acute myeloid leukemia: determination of prognostic significance of rare recurring chromosomal abnormalities among 5876 younger adult patients treated in the United Kingdom Medical Research Council trials. Blood 2010;116:354–365. 10.1182/blood-2009-11-254441 20385793

[15] Greenberg PL, Tuechler H, Schanz J, Sanz G, Garcia-Manero G, Solé F, et al Revised international prognostic scoring system for myelodysplastic syndromes. Blood 2012;120:2454–2465. 10.1182/blood-2012-03-420489 22740453 PMC4425443

[16] Greenberg P, Cox C, LeBeau MM, Fenaux P, Morel P, Sanz G, et al International scoring system for evaluating prognosis in myelodysplastic syndromes. Blood 1997;89:2079–2088. 9058730

[17] Peng J, Hasserjian RP, Tang G, Patel KP, Goswami M, Jabbour EJ, et al Myelodysplastic syndromes following therapy with hypomethylating agents (HMAs): development of acute erythroleukemia may not influence assessment of treatment response. Leuk Lymphoma. 2016;57:812–9 10.3109/10428194.2015.1079318 26293512

[18] Chen PM, Hsiao LT, Chen MH, Chang PM, Liu CY, Hong YC, et al Current status of hematopoietic stem cell transplantation in Taiwan. Bone Marrow Transplant 2008;42 Suppl 1:S133–s136. 18724286 10.1038/bmt.2008.141

[19] Park S, Picard F, Azgui Z, Viguie F, Merlat A, Guesnu M, et al Erythroleukemia: a comparison between the previous FAB approach and the WHO classification. Leuk Res 2002;26:423–429. 11916513 10.1016/s0145-2126(01)00146-1

[20] Zuo Z, Medeiros LJ, Chen Z, Liu D, Bueso-Ramos CE, Luthra R, et al Acute myeloid leukemia (AML) with erythroid predominance exhibits clinical and molecular characteristics that differ from other types of AML. PLoS One 2012;7:e41485 10.1371/journal.pone.0041485 22844482 PMC3402404

[21] Bacher U, Haferlach C, Alpermann T, Kern W, Schnittger S, Haferlach T. Comparison of genetic and clinical aspects in patients with acute myeloid leukemia and myelodysplastic syndromes all with more than 50% of bone marrow erythropoietic cells. Haematologica 2011;96:1284–1292. 10.3324/haematol.2011.043687 21606170 PMC3166098

[22] Porwit A, Vardiman JW. Acute myeloid leukemia with expanded erythropoiesis. Haematologica 2011;96:1241–1243. 10.3324/haematol.2011.050526 21880638 PMC3166090

[23] Hasserjian RP, Zuo Z, Garcia C, Tang G, Kasyan A, Luthra R, et al Acute erythroid leukemia: a reassessment using criteria refined in the 2008 WHO classification. Blood 2010;115:1985–1992. 10.1182/blood-2009-09-243964 20040759 PMC2942006

[24] Santos FP, Faderl S, Garcia-Manero G, Koller C, Beran M, O'Brien S, et al Adult acute erythroleukemia: an analysis of 91 patients treated at a single institution. Leukemia 2009;23:2275–2280. 10.1038/leu.2009.181 19741728 PMC4217206

[25] Wang SA, Hasserjian RP. Erythroid proliferations in myeloid neoplasms. Hum Pathol 2012;43:153–164. 10.1016/j.humpath.2011.08.008 22154053

[26] Liu W, Hasserjian RP, Hu Y, Zhang L, Miranda RN, Medeiros LJ, et al Pure erythroid leukemia: a reassessment of the entity using the 2008 World Health O4ganization classification. Mod Pathol 2011;24:375–383. 10.1038/modpathol.2010.194 21102413

[27] Wong E, Ling V, Westerman D, Morgan S, Juneja S. How unique is pure erythroid leukaemia? A retrospective analysis of seven cases and review of the literature. J Clin Pathol 2015;68:301–305. 10.1136/jclinpath-2014-202740 25609576

[28] Calvo X, Arenillas L, Luno E, Senent L, Arnan M, Ramos F, et al Erythroleukemia shares biological features and outcome with myelodysplastic syndromes with excess blasts: a rationale for its inclusion into future classifications of myelodysplastic syndromes. Mod Pathol. 2016;29:1541–1551 10.1038/modpathol.2016.146 27562492

[29] Wang SA, Patel KP, Pozdnyakova O, Peng J, Zuo Z, Dal Cin P, et al Acute erythroid leukemia with <20% bone marrow blasts is clinically and biologically similar to myelodysplastic syndrome with excess blasts. Mod Pathol. 2016;29;1221–1231 10.1038/modpathol.2016.118 27443511

[30] Bennett JM, Tuechler H, Aul C, Strupp C, Germing U. Dysplastic erythroid precursors in the myelodysplastic syndromes and the acute myeloid leukemias: Is there biologic significance? (How should blasts be counted?). Leuk Res 2016;47:63–69. 10.1016/j.leukres.2016.05.006 27258735

[31] Devillier R, Gelsi-Boyer V, Brecqueville M, Carbuccia N, Murati A, Vey N, et al Acute myeloid leukemia with myelodysplasia-related changes are characterized by a specific molecular pattern with high frequency of ASXL1 mutations. Am J Hematol 2012;87:659–662. 10.1002/ajh.23211 22535592

[32] Devillier R, Gelsi-Boyer V, Murati A, Prebet T, Rey J, Etienne A, et al Prognostic significance of myelodysplasia-related changes according to the WHO classification among ELN-intermediate-risk AML patients. Am J Hematol 2015;90:E22–24. 10.1002/ajh.23850 25219760

[33] Liu CJ, Hong YC, Yang CF, Liu SH, Gau JP, Liu JH, et al Clinicopathologic features and outcome of acute erythroid leukemia based on 2008 revised World Health Organization classification. Leuk Lymphoma 2012;53:289–294. 10.3109/10428194.2011.607526 21780998

[34] Lessard M, Struski S, Leymarie V, Flandrin G, Lafage-Pochitaloff M, Mozziconacci MJ, et al Cytogenetic study of 75 erythroleukemias. Cancer Genet Cytogenet 2005;163:113–122. 10.1016/j.cancergencyto.2005.05.006 16337853

[35] Kornberg A, Polliack A. Serum lactic dehydrogenase (LDH) levels in acute leukemia: marked elevations in lymphoblastic leukemia. Blood 1980;56:351–355. 6931618

[36] Wimazal F, Sperr WR, Kundi M, Meidlinger P, Fonatsch C, Jordan JH, et al Prognostic value of lactate dehydrogenase activity in myelodysplastic syndromes. Leuk Res 2001;25:287–294. 11248325 10.1016/s0145-2126(00)00140-5

[37] Komrokji RS, Corrales-Yepez M, Kharfan-Dabaja MA, Al Ali NH, Padron E, Rollison DE, et al Hypoalbuminemia is an independent prognostic factor for overall survival in myelodysplastic syndromes. Am J Hematol 2012;87:1006–1009. 10.1002/ajh.23303 23090887

[38] Khan AM, Lancet JE, Kharfan-Dabaja MA, Al Ali NH, List AF, Komrokji RS, et al Albumin as a prognostic factor for overall survival in newly diagnosed patients with acute myeloid leukemia (AML) [abstract]. Blood 2011; 118: (Supplement) Abstract 4253

